# Spatial Modeling of Insect Pollination Services in Fragmented Landscapes

**DOI:** 10.3390/insects15090662

**Published:** 2024-08-30

**Authors:** Ehsan Rahimi, Chuleui Jung

**Affiliations:** 1 Agricultural Science and Technology Institute, Andong National University, Andong 36729, Republic of Korea; ehsanrahimi666@gmail.com; 2 Department of Plant Medical, Andong National University, Andong 36729, Republic of Korea

**Keywords:** insect pollinators, pollination services, fragmented landscapes, forest patches, landscape metrics, Lonsdorf model

## Abstract

**Simple Summary:**

This study focuses on improving the understanding of how forest fragmentation impacts pollination by using a modified version of the Lonsdorf model, which better accounts for bee movement in agricultural landscapes. The researchers created simulated landscapes with varying proportions of forest and degrees of fragmentation. We found that increased forest fragmentation, characterized by smaller and more isolated patches, can enhance pollination services due to greater nesting suitability and nearby floral resources. The findings emphasize the importance of using different models for pollination mapping, as the modified model provides unique insights compared to the original Lonsdorf model.

**Abstract:**

Pollination mapping and modeling have opened new avenues for comprehending the intricate interactions between pollinators, their habitats, and the plants they pollinate. While the Lonsdorf model has been extensively employed in pollination mapping within previous studies, its conceptualization of bee movement in agricultural landscapes presents notable limitations. Consequently, a gap exists in exploring the effects of forest fragmentation on pollination once these constraints are addressed. In this study, our objective is to model pollination dynamics in fragmented forest landscapes using a modified version of the Lonsdorf model, which operates as a distance-based model. Initially, we generated several simulated agricultural landscapes, incorporating forested and agricultural habitats with varying forest proportions ranging from 10% to 50%, along with a range of fragmentation degrees from low to high. Subsequently, employing the modified Lonsdorf model, we evaluated the nesting suitability and consequent pollination supply capacity across these diverse scenarios. We found that as the degree of forest fragmentation increases, resulting in smaller and more isolated patches with less aggregation, the pollination services within landscapes tend to become enhanced. In conclusion, our research suggests that landscapes exhibiting fragmented forest patch patterns generally display greater nesting suitability due to increased floral resources in their vicinity. These findings highlight the importance of employing varied models for pollination mapping, as modifications to the Lonsdorf model yield distinct outcomes compared to studies using the original version.

## 1. Introduction

Analyzing FAO data spanning from 1961 to 2006, Aizen et al. (2009) identified 87 crops reliant on pollinators. Their analysis suggested that the absence of pollinators could lead to a decline of 3–8% in global crop production [1]. Over the period from 1961 to 2006, both the production and consumption of pollinator-dependent crops witnessed global growth, particularly in developed countries (a 16.7% increase compared to 9.4% in developing countries) [1]. The presence of pollinators stands out as a particularly important factor for the production of pollinator-dependent crops [2,3,4]. Among pollinators, insects, such as moths, butterflies, bumblebees, honeybees, solitary bees, and hoverflies, play a pivotal role [5]. Bees emerge as the most vital pollinators, contributing significantly to global crop production as honeybees and bumble bees collectively visit over 90% of the world’s food crops [6].

The recent decrease in bee populations [7], the potential effects of climate change on bees [8], and the increasing demand for pollination services in agricultural landscapes have prompted a consideration of strategies to attract bees and other pollinators in these landscapes. In agricultural landscapes, pollination hinges on the movement of native pollinators from non-agricultural areas such as forests (nesting habitats) to farms (foraging habitats) [9]. The arrangement of the habitat patches, especially how close these habitats are to each other, significantly influences the efficiency of pollination [10]. Various studies have demonstrated the influence of forest patches on the abundance of bees within these landscapes [11,12]. Therefore, one strategy involves enhancing pollination services in the agricultural landscape by creating new natural patches as nesting habitats (such as forests) for bees in appropriate locations [13,14,15,16]. In this regard, understanding these dynamics is pivotal for implementing targeted measures that promote the well-being and proliferation of pollinators in diverse and interconnected ecosystems [16].

Rahimi, et al. [11] also conducted a review of 93 articles investigating the impact of forest patches on bees in agricultural landscapes. Their analysis revealed that approximately 79% of these studies indicated a decrease in both bee diversity and abundance with an increase in distance from forest patches, up to a range of 2 km. This trend was observed regardless of various factors such as species type, surrounding agricultural land use, and characteristics of the forest patches (e.g., size and number). Moreover, about 76% of the studies highlighted a positive correlation between the presence of forest cover within a 2 km radius of the study sites and bee populations. In another study, Ulyshen, et al. [12] demonstrated that various pollinator taxa heavily rely on or greatly benefit from resources found exclusively in forests, including floral resources provided by forest plants (including wind-pollinated trees), nesting materials like dead wood and tree resins, and non-floral sugar sources such as honeydew.

Extensive evidence across multiple crop types suggests that forest cover can significantly boost yields in adjacent habitats, particularly within the foraging ranges of pollinators. Moreover, they found that forests may assume an even greater importance for pollinators in the future due to their potential role in mitigating the adverse effects of pesticides and climate change [15]. Nonetheless, many questions persist regarding the optimal amount and configuration of forest cover necessary to enhance the diversity of forest-associated pollinators and their services, both within forests and in neighboring habitats.

The majority of studies in this field have primarily concentrated on the combined consequences of habitat loss and fragmentation. As a result, researchers sometimes encounter contradictory findings [17,18,19]. Although habitat loss and fragmentation frequently occur together, they have distinct ecological implications. Habitat loss refers to a reduction in the overall amount of habitat, potentially leading to its complete disappearance if the trend persists. Nonetheless, it is vital to differentiate the concept of fragmentation per se, which pertains to fragmentation regardless of the overall habitat amount (no habitat loss) in a landscape, where the alterations occur solely in the arrangement of patches [20]. Hence, in scenarios where the total habitat remains constant, but the level of fragmentation varies, the advantages for biodiversity tend to favor a fragmented landscape structure. For instance, Fahrig [21] reviewed 381 studies concerning the effects of fragmentation, per se, on biodiversity. Their analysis revealed that 290 (76%) of these studies reported positive effects of fragmentation per se.

Indeed, in practical terms, investigating the impacts of habitat loss, fragmentation per se, or the establishment of new forest patches in an agricultural setting can be challenging and time-intensive. Therefore, simulation-based studies are often recommended [22]. These studies utilize mechanistic models to describe the movement of pollinators within agricultural landscapes and can explore a variety of scenarios involving habitat loss and fragmentation [13,14,23,24,25]. By employing simulations, researchers can systematically assess the potential outcomes of different landscape configurations, providing valuable insights into the dynamics of pollinator behavior and the effects of landscape alterations.

For instance, Mitchell, et al. [24] discovered that landscapes with moderate levels of habitat and fragmentation provide the highest pollination services.

Introduced in 2009 by Lonsdorf and collaborators [26], the widely adopted model for predicting pollinator numbers in a landscape emphasizes the return of wild bees to nests after collecting pollen and nectar. The model predicts bee abundance in agricultural fields based on distance between nesting habitats and floral patches. While assuming natural habitats provide nesting spaces and surrounding areas are for foraging, the model correlates pollinator abundance with pollination level. Nowadays, the process of mapping pollination continues to rely on the foundational Lonsdorf model within the InVEST_3.14.2_ software [27]. Based on this model, Rahimi, et al. [14] investigated how pollination would change with habitat loss and fragmentation. They discovered that, at the landscape level encompassing both forests and farms, fragmentation per se led to a decrease in pollination services. However, at the farm level, landscapes with the highest degree of fragmentation per se exhibited the maximum pollination service.

Regarding different models for pollination mapping, a review study [28] analyzed 42 research efforts that developed non-correlative models to investigate how land use and land cover changes affect bee populations. The review synthesized information on the modeled systems, methodologies, and key model features such as spatiotemporal scope and resolution. Various modeling approaches are used to predict bee biodiversity and the pollination services they provide, with a greater focus on wild populations compared to managed ones. Among these models, landscape indicators and distance decay models are relatively straightforward, involving fewer parameters. These models facilitate the mapping of bee visitation probabilities using basic land cover data and considering bee foraging ranges.

However, various studies have highlighted and discussed the limitations of this model [29,30,31]. For instance, one limitation is that the model assigns higher scores of nesting suitability to the interior parts of forest patches compared to the margins. Ecologically, it is expected that bees would choose marginal and edge areas as suitable nesting habitats, especially if the patch is very large. Bees are considered central place foragers (CPF), meaning they weigh the cost of traveling versus the rewards obtained from far patches in the landscape (Bell, 1990). In CPF-based models, bees do not visit patches beyond their foraging distance. For example, Zulian*,* et al. [32] adopted an applied methodology known as the ESTIMAP model (Ecosystem Services Mapping at the European Scale) to map the pollination ecosystem across Europe. This methodology maintained the forest edge score constant, while the core area score decreased from its edge toward its center. A similar study followed this methodology at a national level in Iran [33].

Hence, by modifying the Lonsdorf model to prioritize forest edges over interior parts, our comprehension and interpretations concerning the effects of forest fragmentation on pollination services may undergo substantial transformation. Such adaptation promises a more accurate portrayal of these effects. While Rahimi, et al. [33] explored the effects of fragmentation on pollination using the original model, this study examines analogous inquiries through the lens of the modified model. The principal aim of this current investigation is to elucidate how a modification to the Lonsdorf model can reshape our understanding of the influence of forest patch configurations on pollination services.

## 2. Materials and Methods

### 2.1. Generating Simulated Landscapes

**Figure 1:**
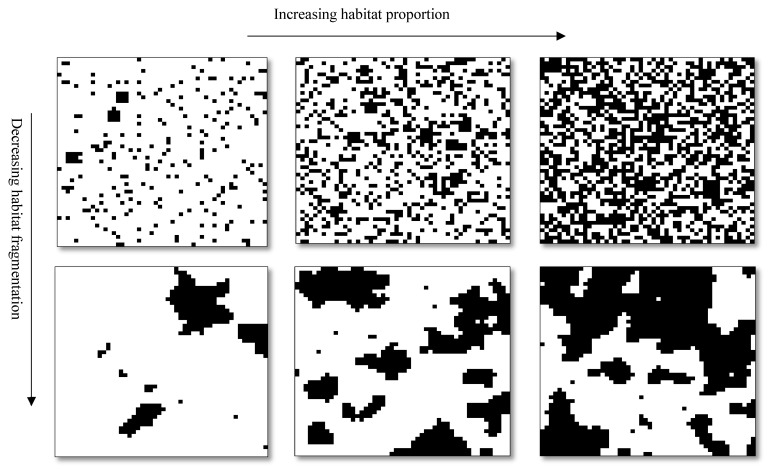
Simulated landscapes in different forest proportions (black patches) and degree of fragmentation.

Figure 1 visually represents some of the simulated landscapes created for our study, which primarily delves into landscapes hosting multiple forest patches amid a matrix adorned with flowers. In crafting these landscapes, we used the “nlm_randomcluster” function in the NLMR package [34] in R v4.3 software. This function simulates a random cluster nearest-neighbor-neutral landscape. In this function, the parameter “*a*” regulates the diversity and abundance of land cover classes, while “*p*” governs the proportion of elements randomly chosen to compose clusters. We created 50 × 50 cell landscapes where forest patches coexist with farms or pastures featuring diverse flowering plants. Notably, we manipulated forest coverage and fragmentation levels across various scenarios. Forest proportion varied from 10% to 50%, yielding five sets of simulated landscapes representing distinct forest proportions (10, 20, 30, 40, and 50% of the entire landscape). Simultaneously, the degree of fragmentation, controlled by the parameter “*p*” in the NLMR package, spanned from the highest (0.01) to the lowest (0.55). A fragmentation degree of 0.01 denotes landscapes with maximal fragmentation, resulting in highly fragmented patterns of forest patches. Our process involved generating 24 different *p* values within each forest cover scenario, resulting in a total of 100 maps generated for each *p* across the five forest cover scenarios. Ultimately, we created 12,000 (5 × 24 × 100) simulated landscapes, where the average intensity across the 100 images was calculated for each p scenario.

### 2.2. Assigning Bees and Flowers to Simulated Landscapes

**Figure 2:**
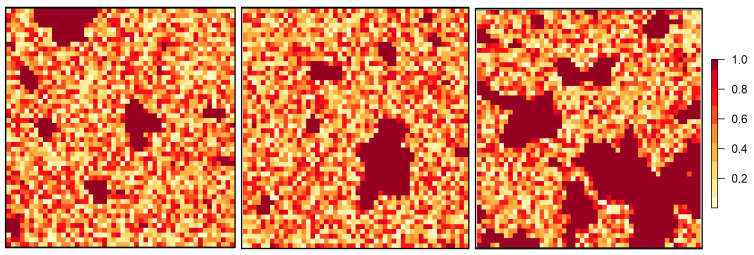
Examples of simulated landscapes after assigning bees and flowers to cells. Cells with a value of 1 represent forested areas, depicted by the brown color. Conversely, cells designated as farm cells receive a random value between 0 and 0.9. A value of 0 indicates the absence of floral resources, whereas a value of 0.9 signifies the highest desirability of floral resources for bees.

In this study, the simulated landscapes are structured with a binary framework, where each cell is represented by either a one (forest patches) or a zero (floral resources). We randomly assign values between 0 and 0.9 to all cells that correspond to floral resources. The premise is that bees primarily inhabit forest patches as nesting sites, and from these patches, they move towards the randomly distributed floral resources across the landscape. These initial landscapes serve as the fundamental framework for all subsequent modeling efforts (Figure 2). While we recognize the complexity of floral resource distribution, including both farmland and forest areas [35,36,37], our modeling was limited to floral resources in farmland to maintain consistency in our scenario-based approach. Including forest cells as potential flower locations would change the proportion of forest cover, impacting the study’s scenarios.

We hypothesized that insect pollinators could visit flowers within a radius of up to five cells from their position, with each cell representing a 400 × 400 m area. This translates to an average foraging range of 2 km for *Apis mellifera,* for example [38]. In a review study, Zurbuchen et al. [39] investigated the furthest distances bees travel while foraging. They discovered that solitary bees typically forage up to 1220 m, bumblebees up to 14,670 m, stingless bees up to 1520 m, and honeybees up to approximately 6313 m. To simulate this, we assigned each cell in a farm a unique numerical value representing a distinct flower species (Appendix A).

Our simulated landscapes each contained 2500 cells, with a designated portion as forest patches. The number of flowers was determined by four scenarios, varying the extent of farmland in each landscape. For instance, in a landscape with 250 forest cells, where 10% were covered by forest, we assigned 225 unique flower species to the 2250 farmland cells. Each scenario’s outcomes are unique and not directly comparable, as different numbers of flowers were assigned to each (Table 1). 

### 2.3. Modeling Pollination

We used a modified version of the Lonsdorf model [29,30] for nesting suitability mapping with two conditions as follows: first, bees were limited to flights spanning a maximum of five cells; second, for assessing nesting suitability, only floral resource values were considered. In this scenario, against the Lonsdorf model [26], edge cells receive higher values than central cells. The output values for nesting suitability from Equation (1) range from 0 to 1.

(1) $$G_{i}=N_{i}\frac{\sum_{j=1}^{M}F_{j}e^{{-D}_{ij/\alpha }}}{\sum_{j=1}^{M}e^{{-D}_{ij/\alpha }}}$$

The equation assesses habitat suitability for the target species’ nesting, assigning *Ni* a value of 1 if suitable and 0 otherwise. *Dij* represents the Euclidean distance between nesting (*i*) and floral resource (*j*) cells. The numerator aggregates weighted distances from adjacent floral cells near nesting patches, with cell quality (*Fj*) ranging from 0 to 1. In the Lonsdorf model, α denotes the typical bee travel distance from forests to agriculture; we set α at five pixels for our study.

For computing bee abundance on floral resources, a method akin to Equation (1) is employed. Farm cells in proximity to nesting habitats receive higher probabilities for hosting wild bee abundance. The abundance index of P in farm cells (j) is thus calculated using Equation (2), with *Gi* denoting the nesting patch fitness from the initial step (Appendix A).

(2) $$P_{j}=\frac{\sum_{j=1}^{M}G_{i}e^{{-D}_{ij/\alpha }}}{\sum_{j=1}^{M}e^{{-D}_{ij/\alpha }}}$$

### 2.4. Landscape Metrics

To assess the impact of landscape structure on pollination, we employed landscape metrics in Fragstats v4.2 [40]. Six key metrics were computed for all simulated landscapes at the class levels (Table 2). The Mean Patch Area (Area-MN) metric calculates the average size of patches, with higher values indicating larger and potentially less fragmented patches. Conversely, the Edge Density (ED) metric measures the proportion of edge habitat relative to the total landscape area, where higher values signify greater fragmentation due to increased edge habitat. The Aggregation Index (AI) quantifies patch distribution, with lower values suggesting more dispersed patches and higher values indicating clustered patches. Similarly, the Landscape Division Index (DIVISION) captures the spatial heterogeneity or division among patches, with higher values indicating increased fragmentation. The Number of Patches (NP) metric simply counts the total number of patches, with higher counts indicating higher fragmentation. Finally, the Euclidean Nearest-Neighbor Distance (ENN) measures the average distance between patches, where lower values suggest less fragmentation due to closer patch proximity and higher values indicate greater fragmentation with patches farther apart [41].

Initially, we conduct a correlation analysis among landscape metrics, as they often exhibit significant correlations. Subsequently, we opted to utilize the Number of Patches (NP) metric as a representative indicator for the broader set of landscape metrics and perform a linear regression analysis. In this regression, pollination service serves as the dependent variable, and landscape metrics act as independent variables.

## 3. Results

### 3.1. Pollination Mapping

**Figure 3:**
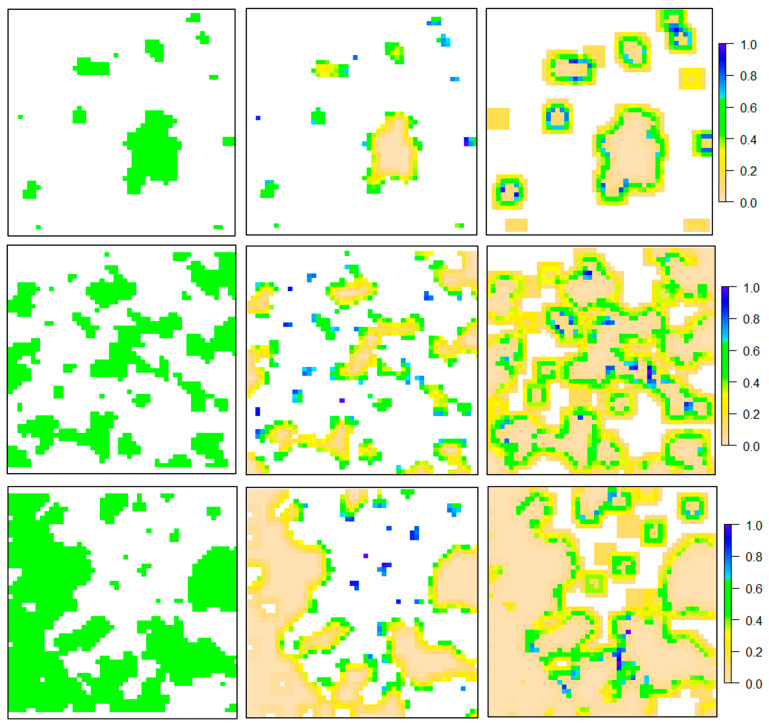
Predicted pollination outcomes utilizing the modified Lonsdorf model amidst diverse fragmentation and forest proportion patterns. In the first column (**A**), the original landscapes are depicted, where green patches represent forest patches. The subsequent column (**B**) illustrates the fitness of these patches based on surrounding floral resources. The third column (**C**) presents the final pollination maps, spanning the entire landscape and employing a 5 by 5 window. The rows in the figure illustrate variations in forest proportion and the degree of fragmentation. It is important to note that these landscapes were chosen merely as examples to demonstrate the concept.

Figure 3 depicts estimated pollination outcomes using the modified Lonsdorf model across diverse fragmentation patterns. In Column B, results from Equation (1) (as explained in the methodology section) showcase the nesting suitability of forest patches concerning nearby floral resources. The illustration highlights that smaller patches and edge cells exhibit increased nesting values, attributed to the higher abundance of floral resources surrounding these cells, contrasting with central cells. Column C presents outcome maps from Equation (2), representing a pollination map. Importantly, only cells with floral resources receive values in this illustration, with nesting patches assigned a value of zero. The column underscores that floral cells near nesting patches receive higher values compared to those located at a distance.

**Figure 4:**
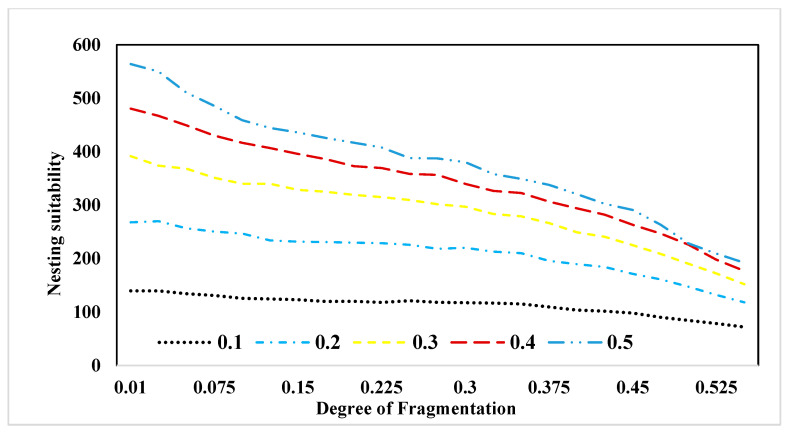
Sum of nesting suitability values for each landscape at different forest fragmentation and proportions.

**Figure 5:**
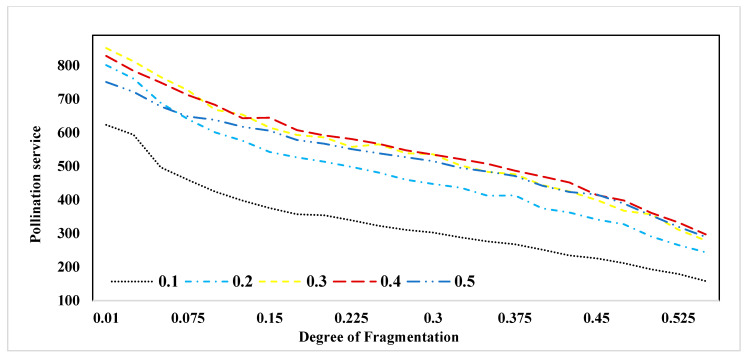
Sum of pollination service values for each landscape at different forest fragmentation and proportions.

Figure 4 displays the cumulative nesting suitability values for each landscape across diverse forest proportions. It is important to note that the degree of fragmentation is represented by values ranging from 0.01 to 0.525, where 0.01 indicates the highest level of fragmentation, and 0.525 represents the highest level of forest patch aggregation. Referring to Column B in Figure 3, nesting suitability maps were previously illustrated. In Figure 4, we aggregate nesting suitability values under distinct forest fragmentation scenarios and proportions. This representation emphasizes that fragmented patterns of forest patches can result in higher suitability values compared to aggregated patterns. Moving to Figure 5, it presents the total sum of pollination values across varying forest proportions and fragmentation patterns. This figure affirms that pollination services demonstrate elevated levels within fragmented landscapes, consistently observed across all forest proportions and fragmentation scenarios.

### 3.2. Correlation Analysis

Table 3 presents Pearson correlation coefficients between various landscape metrics computed with a habitat proportion of 50%. Each cell in the table represents the correlation coefficient between two landscape metrics, ranging from −1 to 1. A value of 1 indicates a perfect positive correlation, −1 indicates a perfect negative correlation, and 0 indicates no correlation. Upon examining the correlation coefficients, we observe several pairs with correlation values close to 1, suggesting strong associations between these metrics. Notably, the pairs with the lowest correlation coefficients include PD and DIVISION with a coefficient of 0.63, LPI and ENN_MN with a coefficient of −0.52, LPI and DIVISION with a coefficient of −0.69, and ED and ENN_MN with a coefficient of −0.71. These findings imply that these pairs of landscape metrics exhibit relatively weaker correlations compared to others in the table, indicating potentially independent aspects of landscape structure at the particular habitat proportion.

### 3.3. Landscape Structure and Pollination Service

Due to the high correlation between the number of patches (NP) with other landscape metrics in Table 3, we opted for this as a representative metric to conduct a regression analysis with pollination. Table 4 presents linear regression equations between the number of patches (NP) and pollination across different forest proportions. Each row in the table corresponds to a specific forest proportion, indicating the percentage of forest cover within the landscape. The “Equation” column displays the regression equations derived from the linear regression analysis. These equations provide insights into the relationship between the number of patches and pollination. The coefficients accompanying the number of patches signify the magnitude of its influence on pollination. For example, at a forest proportion of 0.1, the regression equation suggests that pollination (P) is positively influenced by the number of patches (NP), as indicated by the positive coefficient (0.000163). This implies that as the number of patches in the landscape increases, pollination tends to increase as well. The R-squared values, ranging from 50% to 97.5%, represent the proportion of variance in pollination explained by the regression model. The corresponding p-values, all reported as 0.00, indicate that the observed relationships are statistically significant.

**Figure 6:**
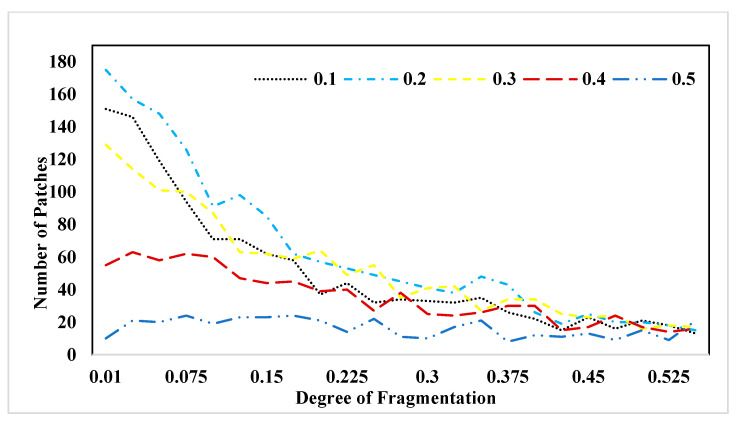
Behavior of the number of patches (NP) metric in different forest proportions and fragmentation.

**Figure 7:**
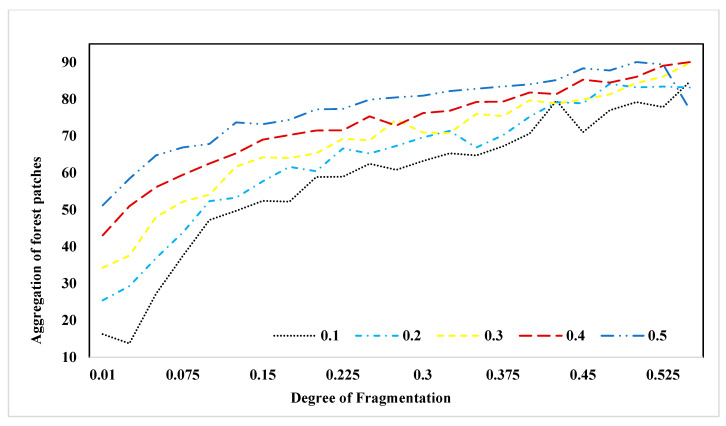
Behavior of aggregation (AI) metric in different forest proportions and fragmentation.

To enhance our comprehension of the relationship between landscape structure and pollination service, we constructed visual representations of two landscape metrics in response to changing fragmentation patterns. In Figure 6, we present the behavior of the “number of patches” (NP) metric across various forest proportions and levels of fragmentation. As indicated by this figure, the number of patches tends to decline as the degree of fragmentation decreases. This illustration highlights that landscapes featuring a forest cover of 20% exhibit the highest number of patches, while those with 50% forest cover demonstrate the lowest count. In Figure 7, we depict the behavior of the “aggregation” (AI) metric across varying forest proportions and degrees of fragmentation. This figure reveals a trend where, as fragmentation decreases, the level of patch aggregation increases. In other words, patches tend to become larger and more closely connected as fragmentation decreases.

## 4. Discussion

This research utilized a modified version of the Lonsdorf model to map pollination across five forest cover scenarios ranging from 10% to 50%, each including various degrees of fragmentation. In the subsequent section, to enhance comprehension of the association between pollination and forest patterns, landscape metrics were employed due to their significant interrelation. Specifically, the number of patches (NP) metric was chosen as a representative of others. The findings indicated a positive correlation between pollination and the number of patches across all habitat proportions, suggesting an increase in pollination with a higher number of patches. The negative correlation observed between this metric and the aggregation metric (AI) suggests a comparable conclusion regarding the impact of fragmentation on pollination.

This trend signifies that as the degree of fragmentation increases, resulting in smaller and more isolated patches with greater aggregation, the pollination services within such landscapes tend to become enhanced. In other words, these fragmented landscapes exhibit a notable propensity to foster heightened pollination services. Based on the modified version of the Lonsdorf model, we found that both pollination and nesting suitability increase as fragmentation increases. This outcome is rooted in a fundamental principle. When a designated level of forest cover is present within a landscape, a fragmented configuration of forest patches translates to their dispersion throughout the landscape. This dispersal signifies that each segment of the landscape stands to gain advantages from these scattered patches. This phenomenon elucidates the reasoning behind the numerous studies that highlight the positive effects of fragmentation per se [21].

This study is simulation-based, making direct comparisons with experimental studies challenging due to the complexity of finding landscapes with fixed forest cover areas while there are varying degrees of fragmentation per se. However, several simulation studies exist for potential comparison, and future endeavors will include contrasting these findings with experimental research results in the Section 4. The study conducted by Rahimi, et al. (2021) [14] serves as the most pertinent comparison for our research, which utilized the Lonsdorf model on a similar dataset of simulated landscapes. This enables us to investigate how modifications to this model could potentially change our understanding of the effects of forest patch fragmentation on pollination. They found that in landscapes with minimal fragmentation, the nesting habitat suitability increases. In contrast, our findings suggest a decrease in suitability under similar conditions (see Figure 4). This difference in findings can be attributed to the significant influence of the distribution of floral resources surrounding patches in determining their suitability, where scattered patches tend to gain more scores.

Findings based on incorporating Lévy flight behavior to study honeybee flower visitation rates in fragmented landscapes [38] are consistent with our findings as they found that the highest average number of visited flowers occurred in landscapes with maximum fragmentation. In areas with less forest cover and greater fragmentation, honeybees visited more flowers due to the increased likelihood of encountering flower cells. Thus, honeybee visitation rates in agricultural landscapes are significantly affected by the degree of forest fragmentation. Other studies have examined the impact of forest patch fragmentation on pollination using different models, such as Mitchell’s model [24]. For instance, Rahimi, et al. [13] applied the model to a dataset of simulated landscapes with different forest proportions and degrees of fragmentation. They found that when the capacity of small patches to supply pollination was limited, fragmented patterns of forest patches led to decreased pollination. However, as the capacity increased, landscapes with a higher degree of forest fragmentation exhibited the highest levels of pollination. Our results also align with Brosi, et al. [23], illustrating how a substantial degree of habitat fragmentation can enhance pollination rates.

Experimental studies frequently yield conflicting results regarding the impacts of fragmentation on pollinators and pollination due to the aforementioned constraints and limitations. For example, Maurer*,* et al. [42] found varying effects of fragmentation, negatively influencing bumblebee colony size in landscapes with minimal habitat coverage but proving beneficial in high-coverage scenarios. Hermansen, et al.’s [43] research also demonstrated that habitat fragmentation in urban mangrove forests leads to reduced pollinator visitation, fruit production, and recruitment. In landscapes with high isolation of forest patches, Farwig, et al. [18] reported a decrease in pollination success. Likewise, Aguilar Aguilar, et al. [44] found that reducing the patch size and increasing the isolation of patches adversely affected pollination. The inconsistency arises because, in real landscapes, the mentioned researchers could not simultaneously control the amount of forest and its degree of fragmentation. As a result, they were unable to isolate the effects of fragmentation per se and instead assessed fragmentation alongside habitat loss, which is known to negatively impact pollinators.

In contrast to the findings of Joshi, et al. [45], we observed a statistically significant increase in pollination with a higher number of patches (NP), attributing it to the augmented availability of nesting habitats and floral resources within these patches. Similarly, the augmentation in average patch size (AREA-MN) in our study resulted in a corresponding decrease in pollination service, as AREA-MN was negatively correlated with NP metric. However, Eigenbrod [46] discusses that, for various ecosystem services (e.g., pollination, pest control, recreation, and water filtration), the delivery of ecosystem services is contingent upon the juxtaposition of natural and non-natural land cover. Such juxtaposition is only feasible in the presence of some level of loss and fragmentation of natural land cover [19,47]. It is important to note that improved pollination services do not automatically translate to enhanced pollinator diversity. For instance, habitat specialists who are sensitive to edge effects may suffer when their habitats are fragmented into numerous smaller patches [48]. Consequently, while fragmentation, as described in our study, might enhance crop pollination services, it does not necessarily support optimal species conservation. Kleijn, et al. [16] demonstrated this limitation by showing that a small subset of common bee species is responsible for the majority of crop pollination services. Specifically, they found that only about 2% of bee species contribute to nearly 80% of the pollination. Therefore, while fragmentation might boost pollination services on a landscape scale, it is crucial to consider that we observed all pollinators as having uniform foraging ranges and behaviors, without accounting for specialized crop–pollinator interactions. For certain crops, there may be specific pollinator behavior models that need to be considered.

## 5. Limitation of This Study

In this study, we modeled landscapes with two types: farms and forests. We assumed flowers only exist on farms and that forests solely serve as nesting sites for pollinators, starting their foraging from there. However, we overlooked the fact that both canopy and understory vegetation in forests provide flowers for pollinators. Canopy trees, for example, offer significant floral resources, though their availability can vary yearly and influence pollinator populations [37]. For example, in tropical forests, many plants depend on animal pollination, with 54% of fruit plants needing bee pollination [49]. Additionally, while we considered diverse farmlands with flowers, we did not account for the negative impacts of intensive agricultural practices, such as herbicide use, which can diminish essential food sources for bees and potentially affect the reliability of our results [50,51,52]. Furthermore, our study’s focus on specific regions and crop systems may limit the generalizability of our findings to other areas with different agricultural practices or ecological conditions. Lastly, the fixed spatial and temporal resolution of our study may not capture variations in floral resource availability and bee foraging behavior over time and across different scales. Future simulations should include flowers in both farms and forests for more realistic honeybee visitation rates. Additionally, intensive agriculture, particularly herbicide use, negatively impacts floral resources for bees. Future studies should consider these impacts to provide a clearer picture of the challenges pollinators face in agricultural landscapes.

## 6. Conclusions

Our study delved into the intricate connection between forest patches and their impact on pollination. Notably, the negative relationship between the number of patches and pollination service highlights the distinct trends within fragmented forest patch landscapes. This trend suggests that increased fragmentation and subsequent smaller, scattered patches tend to enhance pollination services. Our findings, based on the modified Lonsdorf model, reveal that both pollination and nesting suitability increase as fragmentation increases. This arises from the dispersal of patches across landscapes with certain forest cover levels, reinforcing the positive effects attributed to fragmentation per se. These findings collectively underscore the pivotal role of landscape structure in steering bee movement dynamics and shaping flower visitation patterns and pollination service.

## Figures and Tables

**Table 1:** The number of flowers per unit cell of agricultural landscape relative to forest proportion scenario.

Forest Proportion	No. Flowers
10%	225
20%	200
30%	175
40%	150
50%	125

**Table 2:** Descriptions of the selected landscape metrics. In this table, several metrics related to area and edge, shape, and aggregation categories have been presented, and all of them measure landscape configuration aspects.

Category	Metric	Equation	Range
Area and Edge			
	Mean Patch Area (Area-MN)	$\frac{\sum_{j=1}^{n}x_{ij}}{n_{i}}$	Area-MN > 0
	Edge Density (ED)	$\frac{\sum e_{ik}}{A}\left(10000\right)$	0 ≤ ED, no limit
Aggregation			
	Aggregation (AI)	$\left(\frac{g_{ii}}{max\to g_{ii}}\right)(100)$	0 ≤ AI ≥ 100
	Landscape Division Index (DIVISION)	$\left[1-\sum {\left(\frac{a_{ij}}{A}\right)}^{2}\right]$	0 ≤ DIVISION ≥ 1
	Number of Patches (NP)	$n_{i}$	NP ≥ 1
	Euclidean Nearest-Neighbor Distance (ENN)	$h_{ij}$	ENN > 0

*a_ij_* = area (m^2^) of patch; A = total landscape area (m^2^); *n_i_* = number of class i patches in the landscape; *e_ij_* = total length (m) of edges of patch ij, including landscape boundary; *a_ij_ c* = area (m^2^) within patch ij separated from its boundary by a user-specified buffer width (m); g_ii_ = the number of adjacencies (contiguity) between pixels of patch class i; max g_ii_ = maximum possible number of adjacencies among pixels of patches of class i, h_ij_ = distance (m) from patch ij to the nearest neighboring patch of the same type (class), based on patch edge-to-edge distance, computed from cell center to cell center [40].

**Table 3:** Pearson correlation coefficients for landscape metrics were computed with a habitat proportion of 50%.

	NP	PD	LPI	ED	LSI	AREA_MN	ENN_MN	DIVISION
PD	1							
LPI	−0.61	−0.61						
ED	0.94	0.94	−0.69					
LSI	0.94	0.94	−0.69	1				
AREA_MN	−0.76	−0.76	0.60	−0.87	−0.87			
ENN_MN	−0.52	−0.52	0.55	−0.71	−0.71	0.85		
DIVISION	0.63	0.63	−0.97	0.74	0.74	−0.69	−0.66	
AI	−0.94	−0.94	0.70	−1	−1	0.87	0.71	−0.75

**Table 4:** Linear regression equations between the number of patches and pollination.

Forest Proportion	Equation	R2	*p*-Value
0.1	P = 0.2333 + 0.000163 NP	50%	0.00
0.2	P = 0.2169 + 0.000369 NP	97.2%	0.00
0.3	P = 0.2133 + 0.000438 NP	97.5%	0.00
0.4	P = 0.2117 + 0.000501 NP	93.2%	0.00
0.5	P = 0.2071 + 0.000605 NP	85.1%	0.00

“P” in the equation column represents the abbreviation for “pollination”.

## Data Availability

Examples of simulated landscapes are available at https://github.com/ehsanrahimi666/simulation-landscapes.git accessed on 7 January 2024.

## Appendix A

#R codes for pollination modeling

# Function to calculate FQ

FQ <- function(FA, DA, α) {

numerator <- sum(FA * exp(-DA / α))

denominator <- sum(exp(-DA / α))

FQ_i <- numerator / denominator

return(FQ_i)

}

# Load raster files from a folder

raster_folder <- „path/to/your/raster/folder”

raster_files <- list.files(raster_folder, pattern = „\\.tif$”, full.names = TRUE)

# Initialize an empty data frame to store results

results <- data.frame(RasterFile = character(0), P_average_non_zero = numeric(0), G_average_non_zero = numeric(0))

for (raster_file in raster_files) {

# Read the raster file

r <- raster(raster_file)

r <- 1 - r

raster_data <- r

# Get the cell values as a matrix

raster_matrix <- as.matrix(raster_data)

# Replace 0 cells (flower cells) with random values between 0 and 1

flower_cells <- raster_matrix == 0

num_flower_cells <- sum(flower_cells)

random_values <- runif(num_flower_cells, min = 0, max = 0.99) # Generate random values between 0 and 1

# Set random values to the empty cells in the raster matrix

raster_matrix[flower_cells] <- random_values

# Create a new raster with the modified values

modified_raster <- raster(raster_matrix, xmn = extent(raster_data)[1], xmx = extent(raster_data)[2],

ymn = extent(raster_data)[3], ymx = extent(raster_data)[4], crs = projection(raster_data))

# Example usage with a sample raster

raster_data <- modified_raster

# Define the maximum flight range (α) for the bee

α <- 5.0

# Convert raster values to a matrix

raster_matrix <- as.matrix(raster_data)

# Define the radius for assessing floral resources

radius <- α * max(res(raster_data))

# Get cell size

cell_size <- res(raster_data)

# Get cell centers

cell_centers <- xyFromCell(raster_data, 1:ncell(raster_data))

# Initialize an empty matrix to store FQ values

FQ_matrix <- matrix(NA, nrow = nrow(raster_data), ncol = ncol(raster_data))

# Loop over cell centers to calculate FQ values

for (i in 1:length(cell_centers[, 1])) {

row_idx <- rowFromY(raster_data, cell_centers[i, 2])

col_idx <- colFromX(raster_data, cell_centers[i, 1])

if (raster_data[row_idx, col_idx] == 1) { # Only for nesting habitat cells

row_start <- max(1, row_idx - 2)

row_end <- min(nrow(raster_data), row_idx + 2)

col_start <- max(1, col_idx - 2)

col_end <- min(ncol(raster_data), col_idx + 2)

window_cells <- raster_matrix[row_start:row_end, col_start:col_end]

if (sum(window_cells != 1) > 0) { # Exclude cells with value 1

DA <- sqrt((cell_centers[i, 1] - cell_centers[, 1])^2 + (cell_centers[i, 2] - cell_centers[, 2])^2)

FA <- window_cells[window_cells != 1]

# Calculate the number of nesting cells within the window

num_nesting_cells <- sum(window_cells == 1)

# Calculate the number of floral cells within the window

num_floral_cells <- sum(FA > 0 & FA < 1)

# Calculate the FQ value and adjust it using the penalty and reward factors

FQ_value <- FQ(FA, DA[DA <= radius], α)

FQ_matrix[row_idx, col_idx] <- FQ_value

} else {

FQ_matrix[row_idx, col_idx] <- 0 # All window cells are 1, so FQ is 0

}

}

}

FQ1 <-raster(FQ_matrix)

# Calculate the minimum and maximum values of the FQ_raster

min_value <- cellStats(FQ1, min)

max_value <- cellStats(FQ1, max)

# Convert FQ_raster values to the range [0, 1]

FQ_raster_01 <- (FQ1 - min_value) / (max_value - min_value)

# Plot the rescaled FQ_raster using a reversed color palette for higher values

plot(FQ_raster_01, col = rev(topo.colors(100)))

# Convert FQ_raster_01 values of zero to 0.01 and NA values to zero

FQ_raster_01[FQ_raster_01 == 0] <- 0.01

FQ_raster_01[is.na(FQ_raster_01)] <- 0

G_average_non_zero <- mean(FQ_raster_01[FQ_raster_01 > 0], na.rm = TRUE)

# Define the alpha value for the equation

α <- 5.0

# Get the dimensions of the FQ_raster_01

rows <- nrow(FQ_raster_01)

cols <- ncol(FQ_raster_01)

# Initialize a matrix to store the new calculated values

new_FQ_raster <- matrix(NA, nrow = rows, ncol = cols)

# Define the window size

window_size <- 5

# Loop over each cell in the FQ_raster_01

for (i in 1:rows) {

for (j in 1:cols) {

if (FQ_raster_01[i, j] == 0) { # Check for zeros

# Define the window bounds

row_start <- max(1, i - (window_size - 1) / 2)

row_end <- min(rows, i + (window_size - 1) / 2)

col_start <- max(1, j - (window_size - 1) / 2)

col_end <- min(cols, j + (window_size - 1) / 2)

# Extract the window

window_values <- FQ_raster_01[row_start:row_end, col_start:col_end]

# Calculate the decay factor for the window

decay_factor <- exp(-sqrt((i - row_start)^2 + (j - col_start)^2) / α)

# Calculate the sum of values in the window

sum_window <- sum(window_values * decay_factor)

# Calculate the sum of decay factors in the window

sum_decay <- sum(decay_factor)

# Calculate the new central cell value based on the equation

new_FQ_raster[i, j] <- sum_window / sum_decay

} else {

new_FQ_raster[i, j] <- FQ_raster_01[i, j]

}

}

}

# Convert the new_FQ_raster matrix to a raster object

new_FQ_raster_raster <- raster(new_FQ_raster, xmn = extent(FQ_raster_01)[1], xmx = extent(FQ_raster_01)[2],

ymn = extent(FQ_raster_01)[3], ymx = extent(FQ_raster_01)[4])

# Plot the new_FQ_raster_raster using a reversed color palette for higher values

plot(new_FQ_raster_raster, col = rev(topo.colors(100)))

# Calculate the minimum and maximum values of the FQ_raster

min_value <- cellStats(new_FQ_raster_raster, min)

max_value <- cellStats(new_FQ_raster_raster, max)

# Convert FQ_raster values to the range [0, 1]

new_FQ_raster_raster <- (new_FQ_raster_raster - min_value) / (max_value - min_value)

P_average_non_zero <- mean(new_FQ_raster_raster [new_FQ_raster_raster > 0.01], na.rm = TRUE)

results <- rbind(results, data.frame(RasterFile = basename(raster_file), P_average_non_zero, G_average_non_zero))}

# Save results to an Excel file

output_excel <- „path/to/output/pollination_modeling.xlsx”

write.xlsx(results, output_excel, row.names = FALSE)

# Print a message

cat(„Results saved to”, output_excel, „\n”)
